# Cancer glycomics offers potential biomarkers and therapeutic targets in the framework of 3P medicine

**DOI:** 10.3389/fendo.2022.970489

**Published:** 2022-08-22

**Authors:** Yuna Guo, Wenshuang Jia, Jingru Yang, Xianquan Zhan

**Affiliations:** ^1^ Shandong Key Laboratory of Radiation Oncology, Shandong Cancer Hospital and Institute, Shandong First Medical University, Jinan, China; ^2^ Medical Science and Technology Innovation Center, Shandong First Medical University, Jinan, China

**Keywords:** glycosylation, cancer biomarker, immunochemical method, lectin recognition, mass spectrometry, fluorescence imaging, immunotherapy, 3P medicine

## Abstract

Glycosylation is one of the most important post-translational modifications (PTMs) in a protein, and is the most abundant and diverse biopolymer in nature. Glycans are involved in multiple biological processes of cancer initiation and progression, including cell-cell interactions, cell-extracellular matrix interactions, tumor invasion and metastasis, tumor angiogenesis, and immune regulation. As an important biomarker, tumor-associated glycosylation changes have been extensively studied. This article reviews recent advances in glycosylation-based biomarker research, which is useful for cancer diagnosis and prognostic assessment. Truncated *O*-glycans, sialylation, fucosylation, and complex branched structures have been found to be the most common structural patterns in malignant tumors. In recent years, immunochemical methods, lectin recognition-based methods, mass spectrometry (MS)-related methods, and fluorescence imaging-based *in situ* methods have greatly promoted the discovery and application potentials of glycomic and glycoprotein biomarkers in various cancers. In particular, MS-based proteomics has significantly facilitated the comprehensive research of extracellular glycoproteins, increasing our understanding of their critical roles in regulating cellular activities. Predictive, preventive and personalized medicine (PPPM; 3P medicine) is an effective approach of early prediction, prevention and personalized treatment for different patients, and it is known as the new direction of medical development in the 21st century and represents the ultimate goal and highest stage of medical development. Glycosylation has been revealed to have new diagnostic, prognostic, and even therapeutic potentials. The purpose of glycosylation analysis and utilization of biology is to make a fundamental change in health care and medical practice, so as to lead medical research and practice into a new era of 3P medicine.

## 1 Introduction

Post-translational modifications (PTMs) are chemical modifications of proteins during or after translation (1, 2), which include phosphorylation (3), glycosylation (4), ubiquitination (5), acetylation (6), alkylation (7), nitration (8), etc., according to the functional groups modified (1–8). Of these, glycosylation is the most common type of PTMs, and approximately half of all proteins in the human body are glycosylated (9). Glycosylation is a basic enzymatic modification in which glycans are covalently linked to proteins or lipids under the action of enzymes to form glycoproteins and lipopolysaccharides, respectively (4). Glycoproteins are divided into *N*-linked and *O*-linked glycosylations according to the modification site. *N*-glycosylation covalently modifies *N*-acetylglucosamine (GlcNAc) to the nitrogen atom on the side chain of asparagine (Asn). *O*-glycosylation covalently modifies *N*-acetylgalactosamine (GalNAc) to the oxygen atom of a serine (Ser) or threonine (Thr) residue (4, 9). Glycosylation is the most complex PTM process. The diversity of monosaccharides and their combinations greatly increases the diversity of the glycoproteome (10). Glycosylation affects the spatial conformation, activity, and stability of a protein, which in turn affects its subcellular localization and protein-protein interactions (11). Glycosylation is involved in a series of cancer pathophysiological processes, which offers effective and reliable biomarkers for patient stratification, early diagnosis, and prognostic assessment of cancer patients, and effective therapeutic targets/drugs for targeted prevention, and personalized therapy of cancer, in the framework of predictive, preventive, and personalized medicine (PPPM; 3P medicine).

## 2 Structure and functions of glycosylation

**Figure 1:**
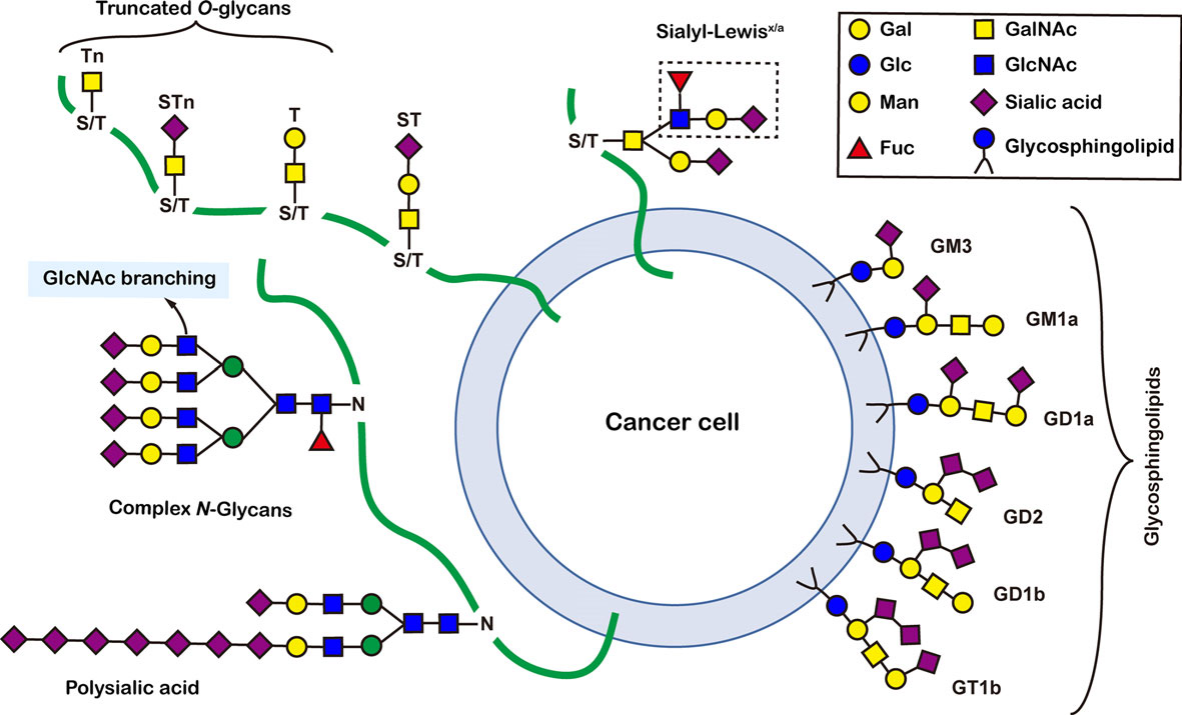
Common glycoconjugates in mammalian cells. *N*-glycans have a pentasaccharide core or a trimannosyl core, and the ends are further modified by GlcNAc, Gal, and SIa. *O*-glycans contain 6 main basic core structures and are further extended to generate structures of various core and different terminal glycans. Glycosphingolipids are composed of ceramides and a series of variable glycans.

There are more than seven thousand configurations of glycan chains in mammals (9). Ten kinds of monosaccharides, namely glucose (Glc), galactose (Gal), mannose (Man), xylose (Xyl), fucose (Fuc), GlcNAc, GalNAc, glucuronic acid (GlcA), iduronic acid (IDOA), and sialic acid (Sia) are the main monomers for glycosylation (4, 9). Glycosylation is a non-templated and highly coordinated process that requires coordination between different glycosyltransferases, glycosidases, nucleotide sugar transporters, and appropriate substrates. Glycosylation changes rapidly with the changes of physiological and pathological conditions (4, 9). Unlike other general types of PTMs such as phosphorylation and ubiquitination that occur in the cytoplasm or nucleus, most glycosylation processes, with the exception of *O*-GlcNAcylation, occur in the endoplasmic reticulum (ER) and the lumen of the Golgi apparatus middle (10). *N*-glycosylations occur in a very large number of proteins and play key roles to regulate many intracellular and extracellular functions. The structural features of *N*-glycans are that they contain a GlcNAc_2_(Man)_3_ core, with the addition or removal of other monosaccharides (**Figure 1**). These additives include Gal, GlcNAc, Sia, and Fuc. *N*-glycosylation in cells starts in ER, and is generally completed in Golgi apparatus. Many glycoproteins have both *N*- and *O*-linked sugar chains. *O*-glycans contain 6 major basic core structures that occur on amino acids with functional hydroxyl groups. *O*-glycosylation takes place in the Golgi apparatus, usually the first linked sugar unit is *N*-GalNAc, and then the sugars are sequentially transferred onto it to form oligosaccharide chains. Glycosylation changes the conformation of polypeptides and increases protein stability (11). Glycosphingolipids are composed of ceramides and oligosaccharide chains, and are common components of eukaryotic plasma membranes. Glycosphingolipids play an important role in cell recognition and communication, especially in the nervous system. In recent years, abnormal glycosylation has received more and more attention in cancer research, mainly in two aspects: (i) Aberrant glycosylation is a non-invasive tumor biomarker, and most FDA-approved tumor markers are glycoprotein or glycan antigens (12). (ii) Glycosylation plays an important role in the occurrence, development, and metastasis of cancer. Researches have shown that glycosylation is associated with cell proliferation, invasion, cell-cell interactions, and cell-matrix interactions (13). In addition, abnormal glycosylation also affects immune regulation (14) and promotes tumor metastasis (15). These have facilitated the development of efficient and innovative analytical methods for glycosylation.

## 3 Aberrant glycosylation in tumors

**Figure 2:**
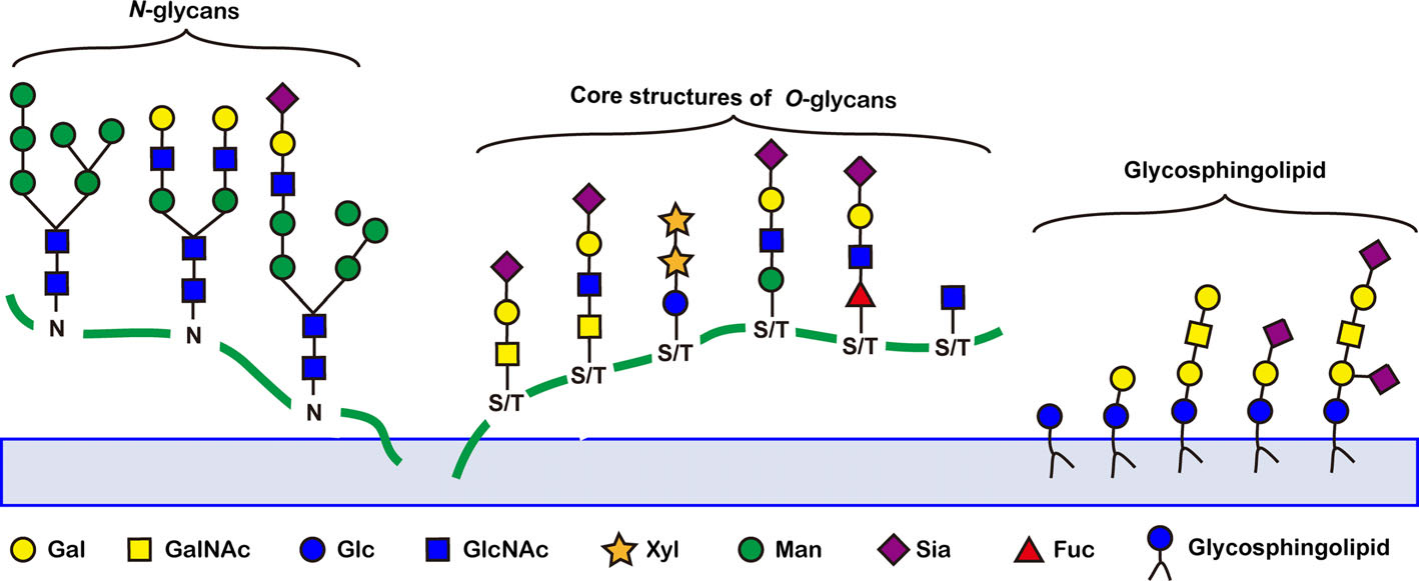
Representative tumor-associated aberrant glycosylation. O-glycan truncation, sialylation, fucosylation, and N-linked glycan branching are abnormally present in cancer and contribute to cancer growth and metastasis.

Abnormal glycosylation is a hallmark of cancer. A number of tumor-associated aberrant glycosylation (**Figure 2**), such as *O*-linked glycan (16–25), sialylation (26–42), fucosylation (43–48), *N*-linked glycan branching (13), are aberrantly present in cancer and contribute to cancer growth and metastasis (**Table 1**). The exploration of aberrant glycosylation that accompanies tumorigenesis and progression began in the 1960s (16, 17). Lectins were used to compare the glycosylation difference between breast cancer and normal cells, which found that tumor cells had stronger binding affinity for lectins, indicating that tumor cells have a higher abundance of specific mucopolysaccharides (17). Mucin is one of the earliest breast cancer serum biomarkers, and truncated *O*-glycans are found in 90% of breast cancers (18). Truncated *O*-glycans, also known as CA72-4 antigen, including Tn (GalNAc-Ser/Thr), T (gal-GalNAc-Ser/Thr), and Sia-Tn (STn, Sia-GalNAc -Ser/Thr) (19). Truncated *O*-glycans are one of the representatives of abnormal glycans in cancer, with increased expressions in gastric (20), pancreatic (21), ovarian (22), bladder (23), and colon cancers (24). STn that is associated with tumor recurrence is considered an important prognostic biomarker, and has been used as a target for antitumor vaccine design (25).

**Table 1:** Representative glycosylated protein or glycan biomarkers in tumors.

Cancer	Biomakers	Sample types	Methods	Effect	Reference
Breast cancer	LY6G6F, VWF, BSG, C1QA, ANGPT1, CDH6	Serum of human	LC-MS	Diagnosis	(12)
Breast cancer	Mucin	Cell of human	Lectin method	Diagnosis	(17)
Breast cancer	Tn	Tissue of mouse	Immunohistological	Functional reseach	(18)
Gastric cancer	Mucin	Tissue of mouse	ELISA	Functional reseach	(20)
Pancreatic cancer	Tn, T, sTn	Tissue of mouse	Immunohistological	Functional reseach	(21)
Ovarian cancer	Tn, T, sTn	Cell of human	Immunohistological	Functional reseach	(22)
Colorectal cancer	Tn	Tissue of human	WB	Diagnosis	(24)
Pancreatic cancer	CA19-9	Tissue of mouse	Immunohistological	Functional reseach	(29)
Colorectal Cancer	CEA, CA19-9	Tissue of human	Public DataBase	Statistics	(30)
Gastric cancer	CEA, CA19-9, CA72-4	Tissue of human	ELISA	Diagnosis	(31)
Metastatic breast cancer	CA15.3, CEA, CA-125, CA19.9	Serum of human	ELISA	Diagnosis	(32)
Gallbladder carcinoma	CA19–9 and CEA	Serum of human	ELISA	Diagnosis	(33)
Breast cancer	Polysialic acid	Tissue of human	HPLC	Diagnosis, functional reseach	(36)
Neuroblastoma	Polysialic acid	Cell of human	WB	Functional reseach	(37)
Cervical cancer	GM1	Serum of human	PCR	Functional reseach	(41)
Breast cancer	GM3	Serum of human	LC-MS	Diagnosis	(42)
Hepatocellular carcinoma	AFP-L3	Serum of human	ELISA	Diagnosis	(44)
Ovarian cancer	Integrins and haptoglobin	Tissue of human	Immunofluorescence	Functional reseach	(45)
Gastric cancer	Haptoglobin	Serum of human	TOF-MS	Diagnosis	(46)
Pancreatic cancer	Fucosylated haptoglobin	Serum of human	L-ELISA	Diagnosis	(47)
Lung cancer	Sialylation, fucosylation	Cell of human	MALDI-TOF MS	Functional reseach	
Lung cancer	Hsp90á	Serum of human	ELISA	Diagnosis	(49)
Liver cancer	AFP	Serum of human	ELISA	Diagnosis	(50)
Breast cancer	AFP	Cell of huma	ELISA	Diagnosis	(51)
Pancreatic cancer	SLex	Tissue of human	Immunofluorescence	Diagnosis	(52)
Pancreatic cancer	MUC6, GlcNAc	Cell and tissue of human	WB	Functional reseach	(53)
Breast cancer	CD82	Tissue of human	Immunohistochemical	Diagnosis	(54)
Lung Cancer	EGFR	Cell	WB	Functional reseach	(55)
Prostate cancer	PSA	Serum and urine of human	L-ELISA	Functional reseach	(56)
Breast cancer	CA15‐3	Serum of human	L-ELISA	Diagnosis	(57)
Breast Cancer	Alpha-1-acid glycoprotein (AGP	Serum of human	ELISA	Diagnosis	(58)
Pancreatic cancer	Sialylation	Tissue of human	Lectin microarray	Functional reseach	(59)
Liver cancer	Tn, á-GalNAc, GlcNAc, Sia	Tissue of human	Lectin microarray	Functional reseach	(60)
Ovarian cancer	Complex N-glycans	Tissue of human	MS	Functional reseach	(61)
Colorectal cancer	Complex N-glycans		MS	Functional reseach	(62)
Triple-negative breast cancer	Polylactosamines	Tissue of human	MS	Functional reseach	(63)
Non-small Cell Lung	Sialylation, fucosylation	Cell of human	MS	Functional reseach	(64)
Colorectal cancer	Carcinoembryonic antigen (CEA)	Tissue of human	MS	Diagnosis	(65)
Liver cancer	GlcNAc, Sialylation,fucosylation	Serum of human	MS	Diagnosis	(66)

By the 1980s, researchers successively discovered that glycosyltransferase activity was differentially expressed between normal and tumor cells. Especially compared to normal cells, tumor cell sialyltransferase and fucosyltransferase activities were significantly increased (26, 27). Abnormally high expressions of Sia and Fuc were quickly recognized as a tumor biomarker due to altered glycosyltransferase expressions. Sialylation is an important modification of cellular glycosylation, and sialylation plays an important role in cell-cell interactions and signal transduction (28). Lewis antigens, including sialyl-Lewis X (SLex, α1,3 fucosylation) and sialyl-Lewis A (SLea, α1,4−fucosylation, also known as CA19-9), are highly expressed in many malignancies (29–33), such as pancreatic (29), colonic (30), gastric (31), breast (32), and biliary tract (33) cancers, and high expression of CA19-9 is associated with poor survival in cancer patients (34). Polysialic acid is an α-2,8-glycosidically linked polymer of Sia, usually expressed as *N*-glycans on neural cell adhesion molecule 1 (NCAM1) (35). The abnormal expression of polysialic acid is associated with the occurrence of lung cancer (35), breast cancer (36), and neuroblastoma (37), and is associated with poor prognosis (38). The increased level of sialylation also involves overexpression of gangliosides (39–42). Gangliosides refer to sialylated glycosphingolipids that are abnormally expressed in tumors such as neuroblastoma (39), lung cancer (40), cervical cancer (41), and breast cancer (42).

Fucosylation plays an important role in tumor pathology, including regulation of signaling pathways and tumor metastasis (43). Aberrant fucosylation has been reported in many cancer types, such as the Lewis antigen is typically associated with tumor progression and metastasis (29–33). The FDA-approved core fucosylated α-fetoprotein (AFP-L3) is widely used as an early diagnosis of hepatocellular carcinoma, and AFP-L3 is more specific than AFP (44). Blood samples from 2447 patients were analyzed for both AFP and AFP-L3. The sensitivity, specificity, and diagnostic odds ratio of AFP and AFP-L3 for hepatocellular carcinoma were analyzed and compared, which found that AFP-L3 had high-specificity and low-sensitivity in diagnosing early hepatocellular carcinoma. It suggests that AFP-L3 could be used to exclude hepatocellular carcinoma in the presence of elevated AFP. Haptoglobin is a protein normally present in the blood that promotes angiogenesis (43). Fucosylated haptoglobin is associated with a variety of diseases, including ovarian (45), gastric (46), and pancreatic (47) cancers. The sensitivity of fucosylated haptoglobin to pancreatic cancer exceeds CA19-9 and CEA (47). When Fuc is highly expressed in epidermal growth factor receptor (EGFR), EGFR dimerization and phosphorylation are increased, and EGFR-mediated tumor cell growth and malignancy-related signaling pathways are increased (48).

## 4 Recent advances in glycomics-based biomarker discovery

Since tumor-associated glycosylation alterations are a distinct feature of cancer diagnosis and prognosis, a great deal of effort has been devoted to identify, label, and characterize glycosylation in recent years. Four types of glycosylation research tools have been developed, including immunochemical methods, lectin recognition-based methods, mass spectrometry (MS)-related methods, and fluorescence imaging-based *in situ* analysis methods. These studies not only focus on the analysis of glycosylation, but also reveal the regulatory mechanism of glycosylation.

### 4.1 Immunochemical methods

**Figure 3:**
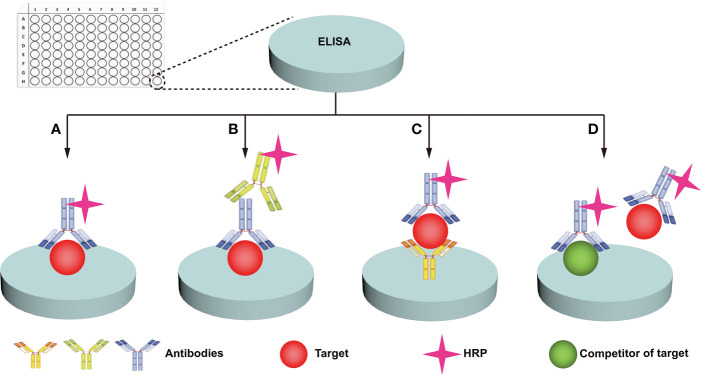
ELISA analysis of protein glycosylation. Four main types of ELISA assays are used: direct **(A)**, indirect **(B)**, sandwich **(C)**, and competition **(D)**.

Immunochemical methods mainly include enzyme-linked immunosorbent assay (ELISA) and western blotting (WB). ELISA uses specific antibodies to specifically recognize and quantify glycans/glycosylated proteins of interest (67). ELISA is widely used in clinical disease diagnosis and is the gold standard (68) for protein detection due to its simple sample pretreatment and quick results. Four main types of ELISAs, including direct, indirect, sandwich, and competition, are used (**Figure 3**) (49, 67, 68). (i) For the direct method (**Figure 3A**), the target is adhered to the well plate, the HRP-labeled antibody binds to the antigen (target), and the enzyme catalyzes the chromogenic substrate to produce a visible colorimetric output, which is measured by a UV-Vis spectrophotometer. The concentration of the analyte is proportional to the intensity of the color. (ii) For the indirect method (**Figure 3B**), the target is adhered to the well plate, the enzyme is bound to the secondary antibody that can recognize the primary antibody, and the signal recognized by the primary antibody is displayed by the secondary antibody. The enzyme catalyzes a chromogenic substrate, and the degree of color development is proportional to the concentration of the analyte. (iii) For the sandwich method (**Figure 3C**), the captured antibody is adhered to the well plate, the antigen in the analyte is bound to the captured antibody, and the HRP-labeled antibody binds to the antigen and catalyzes the coloration of the substrate. Sandwich methods generally have higher sensitivity and specificity than direct and indirect methods. For the competition method (**Figure 3D**), both the target and competitor can bind the HRP-labeled antibody. The more antigens in the sample, the weaker the signal. The antigen concentration in the sample is therefore inversely proportional to the color intensity. A wide range of samples have been analyzed with ELISA, and new markers of tumor progression and prognosis have been discovered (49). Traditional ELISA relies on the chromogenic reaction of substrate and enzyme, which has low sensitivity, and cannot meet the needs of biomarker analysis in complex biological samples (67, 68). Moreover, the enzyme tag used by ELISA is a natural protein, and heat, pH, or chemical induction can make the enzyme lose its catalytic activity, which is not conducive to the stability of the method (**Table 2**). Researchers have developed several strategies to improve the performance of traditional ELISAs, which can be broadly classified into biotechnology-based (50, 69) and nanotechnology-based (51, 68, 70) methods. For biotechnology-based ELISA, blocking agents have been innovatively used to effectively reduce the background noise of ELISA and eliminate various false positive and false negative signals in serum assays (69). Peptides with binding affinity have been designed and utilized to develop recombinant proteins with higher affinity and thermostability to AFP than natural antibodies (50). For nanotechnology-based ELISA, metal-organic frameworks (MOF)@Hemin-Au composite (70) have been utilized to enhance the stability of AFP immunoassays with labeled antibodies. Nanocomplexes can accelerate electron transfer in electrochemical ELISA, and the research achieved high-sensitivity analysis of EGFR (51) and tumor antigen 125 (68) with nanocomplexes, in which the sensitivity of tumor antigen 125 was more than 6 times that of traditional ELISA (68). WB is a traditional protein analysis method to identify and quantify specific proteins in biological samples (48). Tumor biomarkers such as MUC1 (52), MUC6 (53), CD82 (54), EGFR (55), PSA (56), and other glycosylated proteins (71) have been extensively studied with WB. These methods are very sensitive, capable of detecting up to 0.1 nanograms of proteins in a sample (72). Despite its high sensitivity and specificity, WB can still produce incorrect results. For example, false negatives can occur when large proteins do not have enough time to transfer to the membrane. Moreover, processing samples at high temperature may destroy their PMTs (72), which is not conducive to revealing the true state of the samples. Therefore, we hold the view that WB can only be used as an aid in early diagnosis (**Table 2**).

**Table 2:** Advantages and disadvantages of representative glycosylation analysis techniques.

Technical classification	Research methods	Advantages	Disadvantages	References
Immunochemical Methods	ELISA	Simple sample pretreatment; short analysis time; gold standard for clinical diagnosis	Low-Stability of the kit; lack of glycosylation antibodies; inability to obtain glycosylation site and structure information	(49–51, 67–70)
	WB	Ultrahigh sensitivity; high throughput; wide range of applications	Lack of glycosylation antibodies; False negatives may occur; inability to obtain glycosylation site and structure information	(52–56, 71, 72)
Lectin-based method	L-ELISA	Wide variety of lectins; simple sample preparation; short analysis time	Low-Stability of the kit; inability to obtain glycosylation site and structure information	(57, 58, 73, 74)
	lectin blotting	Ultrahigh sensitivity; high throughput; wide application	False negatives can occur; inability to obtain glycosylation site and structure information	(75)
	lectin cytochemistry	Ultrahigh sensitivity; *in-situ* information acquisition; dynamic tracking possible	Inability to obtain glycosylation site and structure information	(76)
	lectin microarray	High dynamic range; ultrahigh sensitivity; high throughput	Inability to obtain glycosylation site and structure information; no in -situ information available	(59, 60, 77–79)
MS	Top-down MS	Simple sample pretreatment; short analysis time; without any digestion; suitable for proteoform analysis Identify glycan structure information	Not suitable for analysis of hydrophobins; difficult to obtain glycosylation sites; low abundance protein signal suppressed; expensive	(80–85)
	Bottom-up MS	Antibody-free; Identify glycosylation site and structure information; wide range of application	Complex sample preprocessing; no *in-situ* information available; expensive	(61–66, 86–115)
Fluorescence imaging		High dynamic range; ultrahigh sensitivity; *in-situ* information acquisition; dynamic tracking possible	Inability to obtain glycosylation site and structure information; expensive	(116–128)

### 4.2 Lectin-based method

**Figure 4:**
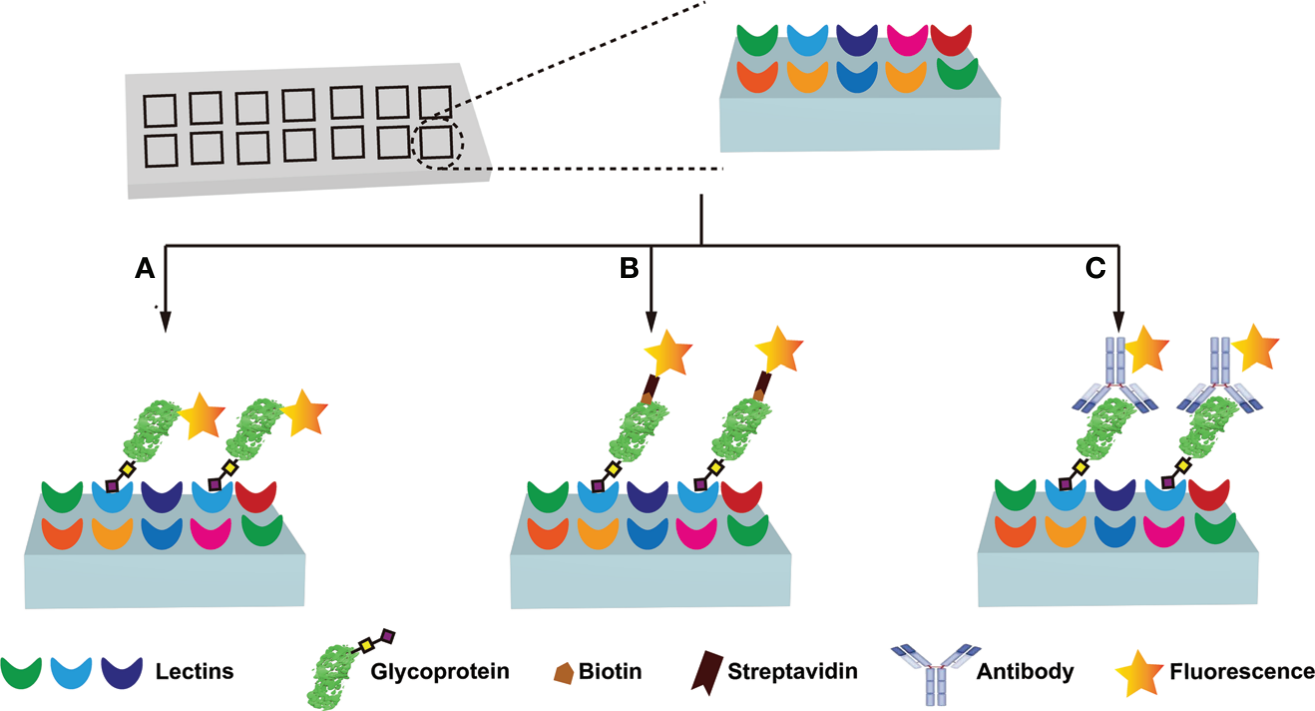
Lectin microarray analysis of protein glycosylation. Three main types of lectin microarray are used: fluorescent labeling of glycoproteins **(A)**, biotin labeling of glycoproteins **(B)**, and antibody recognition of glycoproteins **(C)**.

Immunochemical methods require the use of well-validated antibodies to ensure diagnostic sensitivity and specificity (67, 72). However, the lack of glycan/glycosylated protein-specific antibodies hinders the widespread application of immunochemical methods. Fortunately, lectins have broader specificities, and are considered to be very useful tools for glycan research, and the information on lectins specifically recognizing glycans is summarized (**Table 3**) (57–60, 73–78). Lectin-based methods include lectin-antibody sandwich ELSA (L-ELISA) (67), lectin blotting (67), lectin cytochemistry (67), and lectin microarray (67). L-ELISA and lectin blotting are the extension of ELISA and WB. The principle is the same as that of immunochemical methods, just lectin is used to replace antibody as the recognition molecule (74). L-ELISA has been designed and implemented for the analysis of two breast cancer biomarkers, CA15-3 (57) and α-1-acid glycoprotein (AGP) (58), showing a higher sensitivity than ELISA, distinguishing breast cancer stages I, IIA, and IIB. Lectin blotting has been utilized to explore the link between the development ofliver cancer and the intracellular action of GALNT glycosyltransferase (75). Lectins are widely used for *in situ* tracking of glycan signatures on surfaces; for example, Huang evaluated and compared the glycan structures among 64 cell lines, 37 tissues, and primary colon tumor tissues with 19 fluorescently conjugated lectins (76). Lectin microarray is a fast, sensitive, high-throughput glycan analysis technique suitable for studies with large sample numbers (77) (**Table 2**). Lectin microarrays was used to analyze and compare glycosylation in non-tumor and tumor regions of pancreatic ductal adenocarcinoma (59). Lectin microarray requires less sample, simple pretreatment, and no glycan release or purification, enabling high-throughput, rapid and sensitive glycosylation analysis of different clinical samples. Its analytical methods usually include fluorescent labeling of glycoproteins (**Figure 4A**), biotin labeling of glycoproteins (**Figure 4B**), and antibody recognition of glycoproteins (**Figure 4C**) (59, 77). The most commonly used method is fluorescent labeling of proteins. Briefly, the protein is pre-labeled with a fluorescent dye, and after the glycoprotein is captured by the lectin, the amount of the corresponding glycoprotein is reflected by the fluorescence intensity. Fluorescent dyes such as Cyanine3 and tetramethylrhodamine were widely used (60). Biotin labeling of proteins can further increase the sensitivity of glycosylation assays with streptavidin. However, these two methods have an obvious disadvantage - in order to ensure the reproducibility of the analysis using the direct labeling strategy, a relatively large number of glycoproteins need to be labeled. One way to overcome this deficiency is to employ an antibody-covered lectin microarray strategy, which is more applicable. Increased abundances of sialylated glycans and *N*-GalNAc were found in tumor regions, and the mechanisms underlying these glycosylation-related abnormalities were explored (59). The protein glycosylation changes induced by the drug Sorafenib during tumor therapy have been explored with lectin arrays (78); the high-throughput advantages of lectin arrays were also exploited to systematically analyze the glycosylation of 56 lectins in 207 samples (79). Further, normal and cancerous breast cells were differentiated using lectin microarrays, which have also been used to study cell development and differentiation (60, 74). Another point worth mentioning is that the reversed lectin arrays modify carbohydrates on microarrays, which can be used to analyze carbohydrate-binding proteins such as lectins, and can also be used to study different carbohydrate structures and various biological targets (RNA, virus and whole cell) interactions (60). These studies provided ideas for revealing the molecular mechanism related to glycosylation and designing new anticancer drugs.

**Table 3:** Properties of representative lectins (57–60, 73–78).

Lectin	Abbreviation	Glycoprotein	Metal Ions	Specificity
*Aleuria aurantia*	AAL	No	–	Fucα6GlcNAc
Concanavalin A	Con A	No	Ca^2+^, Mn^2+^	αMan, αGlc
Succinylated Concanavalin A	Succinylated Con A	No	Ca^2+^, Mn^2+^	αMan, αGlc
*Datura stramonium*	DSL	Yes	No	(GlcNAc)_2-4_
*Euonymus europaeus*	EEL	Yes	Ca^2+^, Zn^2+^	Galα3Gal
*Galanthus nivalis*	GNL	No	No	αMan
*Griffonia (Bandeiraea) simplicifolia* I	GSL I, BSL I	Yes	Ca^2+^, Mn^2+^	αGal, αGalNAc
*Hippeastrum* hybrid	HHL, AL	No	No	αMan
Jacalin	Jacalin	Yes	No	Galβ3GalNAc
*Lens culinaris*	LCA, LcH	No	Ca^2+^, Mn^2+^	αMan, αGlc
*Lotus tetragonolobus*	LTL	Yes	Ca^2+^, Mn^2+^	αFuc
*Lycopersicon esculentum*	LEL, TL	Yes	–	(GlcNAc)_2-4_
*Maackia amurensis* I	MAL I, MAL	Yes	No	Galβ4GlcNAc
*Maackia amurensis* II	MAL II, MAH	Yes	No	Neu5Acα3Galβ4GalNAc
*Maclura pomifera*	MPL	No	No	Galβ3GalNAc
*Narcissus* *pseudonarcissus*	NPL, NPA,	No	No	αMan
*Peanut*	PNA	No	Ca^2+^, Mg^2+^	Gaβ3GalNAc
*Pisum sativum*	PSA	Trace	Ca^2+^, Mn^2+^	αMan, αGlc
*Psophocarpus* *tetragonolobus* I	PTL I, WBA I	Yes	–	GalNAc, Gal
*Psophocarpus* *tetragonolobus* II	PTL II, WBA II	Yes	–	GalNAc, Gal
*Ricinus communis* I	RCA I, RCA120	Yes	No	Gal
*Ricin B Chain*	Ricin B Chain	Yes	No	Gal
*Sambucus nigra*	SNA, EBL	Yes	No	Neu5Acα6Gal/GalNAc
*Solanum tuberosum*	STL, PL	Yes	No	(GlcNAc)_2-4_
*Sophora japonica*	SJA	Yes	Ca^2+^, Mn^2+^	βGalNAc
*Soybean*	SBA	Yes	Ca^2+^, Mn^2+^	α>βGalNAc
*Ulex europaeus I*	UEA I	Yes	Ca^2+^, Mn^2+^, Zn^2+^	αFuc
*Vicia villosa*	VVL, VVA	Yes	Ca^2+^, Mn^2+^	GalNAc
*Wheat Germ*	WGA	No	Ca^2+^	GlcNAc
*Succinylated Wheat* *Germ*	Succinylated WGA	No	Ca^2+^	GlcNAc
*Wistera floribunda*	WFA, WFL	Yes	–	GalNAc

### 4.3 MS-based method

**Figure 5:**
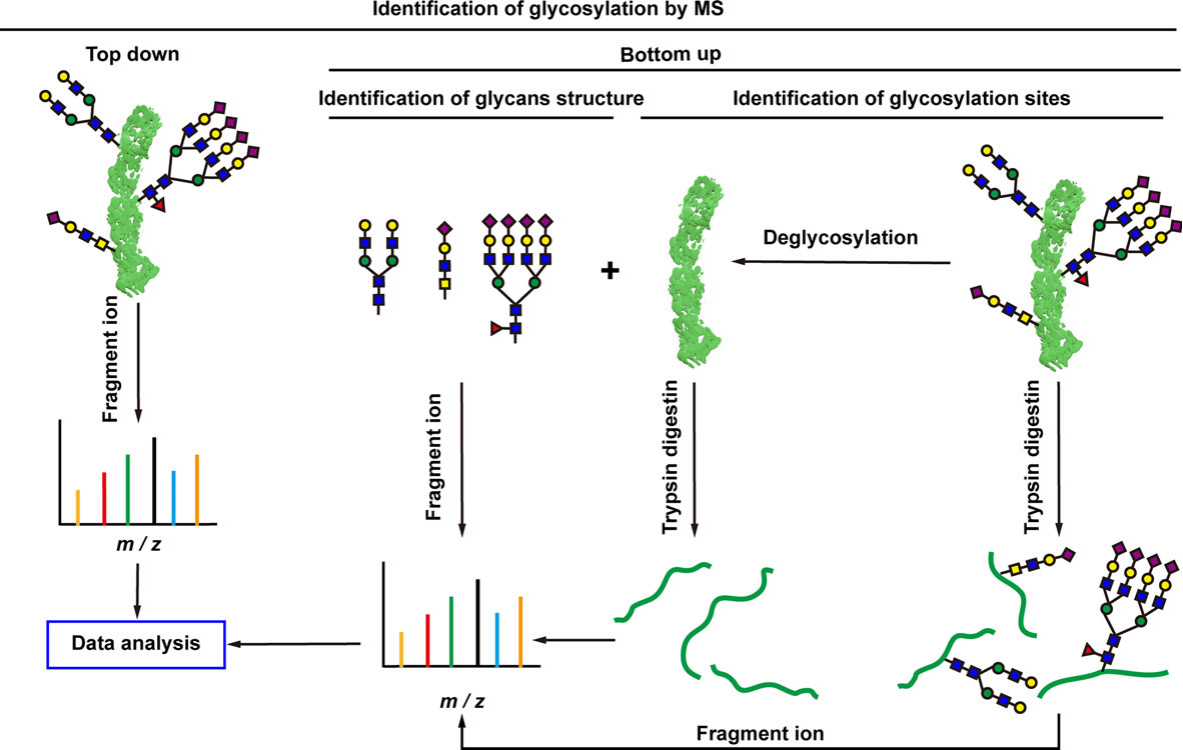
Mass spectrometry analysis of protein glycosylation. Top-down and bottom-up mass spectrometry approaches are used for protein glycosylation analysis. Top-down approach can directly assess the protein backbone and PTMs without any enzymatic digestion prior to MS analysis. Bottom-up approach firstly digests extracted proteins to generate peptides, and then the peptides are used for MS analysis.

Cells are complex living organisms, and subtle changes in glycosylation may transmit different signals and produce different biological effects (129). There is an increasing demand for detailed analysis of the structure and modification sites of glycans. The most significant advantage of MS is the ability to obtain detailed structural information of glycans, which makes MS the best tool for analyzing glycosylations. Matrix-assisted laser desorption/ionization MS (MALDI-MS) and electrospray ionization MS (ESI-MS) are two commonly used MS approaches (130, 131). MALDI is often combined with time-of-flight (TOF) MS, which has a theoretically infinite *m/z* range and fast scan speed, enabling extremely low detection lines. ESI is a form of soft ionization protonation that progressively desolvates the sample and forms analyte ions at lower temperatures. A major advantage of ESI is that it can be easily combined with high performance liquid chromatography (HPLC) to pre-separate complex mixtures of glycans (130, 131). Generally, two types of protein glycosylation MS analyses are used, including top-down and bottom-up (80) (**Figure 5**). Top-down glycoprotein analysis is the direct assessment of PTMs of the protein backbone without any digestion, which has found some new proteoforms (81–84), and some of these proteoforms cannot be reliably identified by bottom-up method (83). It is significant to better understand the molecular mechanisms of a disease. The top-down method is more efficient and rapid for the analysis of glycosylation. The analysis of 38 glycoforms has been achieved in a few hours in combination with bioinformatics tools (82). However, despite the strong potential and technological advancement of the top-down approach, it has rarely seen its widespread and clinical application. Top-down MS study has its own limitations: (i) it is not suitable for the analysis of hydrophobic proteins such as membrane proteins; (ii) it inhibits low-abundance proteins such as glycoproteins, and lack of separation methods for glycosylated proteins; (iii) it is difficult to accurately locate unstable glycosylation modification site (85); and (iv) it requires additional bioinformatics tools to process the complex data generated by top-down approaches (86). These are the problems that need to be solved urgently in the development of top-down MS. Whereas, the bottom-up MS approach digests extracted proteins to generate peptides suitable for MS analysis (87). Glycopeptides are easier to be analyzed with MS than intact glycoproteins because they may exhibit higher ionization efficiencies and yield simpler tandem mass spectrometry (MS/MS) spectra, partialy due to their smaller size than glycoproteins (87). Glycopeptides are often required to be enriched prior to MS/MS analysis, and glycopeptides have relatively low ionization efficiencies compared to non-glycosylated peptides. Analysis of glycan structure requires the release of glycans. *N*-glycans are released from glycopeptides by peptide-*N*-glycosidase F (PNGase F) digestion (61). *O*-glycans have many core structures, and no general release enzymes are used; however, there are corresponding enzymes for specific core structures. Alternatively, any *O*-glycans can be released through the chemical process of β-elimination, with the potential for undesirable side effects (61). In recent years, bottom-up MS has been widely used to study glycosylation changes in tumorigenesis and development at different specificity levels including global, cell-specific, and local-specific.

#### 4.3.1 MS analysis of global glycosylation

MS is widely used in cancer diagnosis and mechanism research. It has been found that *N*-glycan structure is closely related to tumor molecular subtypes, and fucosylation is differentially modified in different subtypes of ovarian cancers (62). MS has been used to characterize changes in glycosylation in sera and tissues from colon cancer patients with stages II and III. Oligosaccharides, hypogalactosylated, and tetra-antennary forms are ur-regulated in tumor tissues (63). The structural distribution of specific types of glycans in the stroma, necrosis, and tumor areas of breast cancer has been studied, and high-Man, branched, and fucosylated glycans were predominantly present in the tumor region (64). More abundant fucosylated and sialylated glycopeptides are found in drug-resistant non-small cell lung cancer cells (65). Researchers also improved MS methods in many ways to obtain more sensitive and comprehensive information. For example, CEA samples purified from human colon cancer and its liver metastastic tissues were cleaved by specific enzymes such as trypsin, intracellular protease gluc, and nonspecific enzyme pronase, respectively; which identified 28 *N*-glycosylations of CEA. Of them, three more *N*-glycosylation sites were identified by gluc digestion than trypsin digestion. This research provides a better understanding of the heterogeneity of CEA glycosylation pattern (88). Virtual multi-stage MS was utilized to simultaneously obtain glycan, peptide sequence, and glycosylation sites. The deglycosylated peptides and intact glycopeptides were mixed for MS analysis. MS^2^ spectra of intact glycopeptides were used to determine glycosyl groups, while MS^2^ spectra of deglycosylated peptides were used to identify peptide backbone sequences. Compared to the traditional multi-stage strategy, the MS^2^ spectrum of deglycosylated peptide can directly recognize the peptide backbone with higher sensitivity (66). Researchers also improved the detection sensitivity and breadth of MS from the methods of glycan labeling (89) and data analysis (90).

#### 4.3.2 MS analysis of membrane protein glycosylation

The cytoplasmic membrane provides a highly interactive platform for intracellular and extracellular information transfer. Proteins on the cell surface are extremely important for the development of tumors (13). Most membrane proteins are glycosylated to regulate life activities such as cell-cell interaction and signal transduction (14). About 70% of FDA-approved drugs target cell surface proteins (91). MS itself cannot distinguish cell surface glycoproteins from intracellular glycoproteins. Compared to the whole cell glycoproteins, cell membrane glycoproteins are less abundant, and are easily confused with intracellular proteins. The enrichment of surface glycoproteins is of great significance for comprehensive analysis by MS. To better investigate the glycosylation on the cell membrane, researchers use density-gradient centrifugation to separate the plasma membrane from the cell, especially sucrose-gradient centrifugation is the most widely used method, which is also a classic method for membrane protein separation (92, 93). However, ultracentrifugation does not separate membrane glycoproteins but all membrane proteins, and this method cannot completely eliminate cytoplasmic proteins (93). In recent years, with the development of chemical biology and MS-based proteomics, more precise analysis of cell surface glycoproteins has become possible.

A typical method for *in situ* labeling of membrane glycoproteins is to oxidize cell surface glycoproteins with sodium periodate, followed by hydrazine chemical capture to enrich the membrane proteins and identify them with MS. A method, cell-surface-capturing (CSC) technology, was first developed by Wollscheid et al. in 2009 for large-scale analysis of surface glycoproteins (94). It optimized the periodate concentration and reaction conditions to maintain cell viability and minimize side-effects. The aldehyde group oxidized by periodate can react orthogonally with biotin hydrazine (BH). After cell lysis, the biotinylated glycopeptides were enriched by streptavidin beads, and then glycopeptides were eluted by PNGase F treatment. CSC enables site-specific analysis of cell surface glycoproteins, which significantly reduces the false-positive rate of surface glycoprotein identification. Characterization of the cell surface proteome of lymphoid malignancies is a first step toward improving personalized diagnosis and treatment of leukemias and lymphomas. The CSC technique was utilized to characterize the cell surface *N*-glycoproteinome of four human malignant lymphocyte cell lines, and a total of 404 cell surface *N*-glycoproteins were identified. Of them, 82 *N*-glycoproteins had not been previously mentioned in the cell surface protein map. Cluster analysis of these MS data was used to reveal the most representative proteins of each cell type, which would facilitate mapping their stages of differentiation, and help identify associated malignancies (95). With the use of this CSC strategy, researchers constructed an extensive database of cell surface proteins. The Cell Surface Protein Atlas (CSPA; wlab.ethz.ch/cspa/) is a public resource containing experimental evidence for cell surface proteins identified in 41 human cell types (96). To improve the performance of CSC technology, researchers made some changes to it, mainly focusing on optimizing the conditions for labeling aldehyde groups, and optimizing peptide enrichment procedures. The bioorthogonal reaction rates of hydrazide and aldehyde groups used in the CSC strategy are slow and inefficient. Aniline was used as a catalyst to increase the labeling rate. Furthermore, biotinylated surface glycoproteins ware enriched at the levels of protein rather than peptide. Biotinylated proteins were enriched with streptavidin-coated beads. Beads were rigorously washed, and bound proteins were trypsinized. The resulting peptide mixture was analyzed by liquid chromatography-MS/MS (LC-MS/MS). As a result, approximately 900 plasma membrane and secreted proteins were identified, including more than 300 transporters and ion channels (97). CSC method requires a large amount of starting material (10^7^ to 10^8^ cells per experiment), possibly due to the large number of sample processing steps resulting in severe loss of samples. Cell surface protein isolation protocols were also optimized to increase cell surface protein coverage. With a special pipette tip, the new workflow is suitable for very small numbers of cells, 10 times less than traditional CSC methods. A total of ~600 cell surface-associated proteins were identified from 1105 cells alone (98).

The oxidative properties of periodate often harm the active state of cells, and labeling conditions based on enzyme-catalyzed cell surface glycoproteins are milder, and the reaction is more efficient (99). Galactose oxidase can specifically oxidize the hydroxyl group at C_6_ position on Gal/GalNAc to an aldehyde group. The reaction rate of galactose oxidase on the cell membrane surface was also improved by the researchers (99). Galactose oxidase releases H_2_O_2_ when it oxidizes glycoproteins. H_2_O_2_ inhibits the activity of galactose oxidase. The authors added HRP to consume H_2_O_2_ at the same time of oxidation, which promoted the completion of the oxidation reaction. The number of identified glycoproteins increased by ~25% after the addition of HRP, and 953 *N*-glycosylation sites within 393 surface glycoproteins were identified in MCF7 cells (99). Combined with quantitative proteomics, ones performed a systematic quantitative analysis of the changes in the surface glycoproteome of breast cancer under drug treatment. The resulting data contribute to a better understanding of the functions of glycoproteins and molecular mechanisms of a disease (99). Moreover, GlcNAc and GalNAc are two common glycosylations, with the same molecular weights and glycosylation sites, which two are difficult to be distinguished with MS. This problem was solved by exploiting the specificity of galactose oxidase. GalNAc can be oxidized by galactose oxidase but GlcNAc cannot. Combined with MS analysis of glycoproteins, 96 Tn antigen-containing glycoproteins were identified in Jurkat cells (100). These data clearly show that this method can clearly distinguish the two glycoforms, mainly due to the specificity of galactose oxidase (100). We believe that this method can be widely used in the biomedical research of Tn antigen. Compared to periodate, galactose oxidase is promising for oxidizing glycans on the cell surface: (i) The reaction is mild, and the oxidation process does not affect cell viability and growth; (ii) With high specificity, the enzyme, a large molecular weight protein, cannot penetrate the cell membrane of living cells, and only extracellular glycans are labeled. This method is suitable for the analysis of surface glycoproteins.

The unnatural sugar metabolism-labeling technology was first proposed by Bertozzi’s team (101). The basic principle is to use the original metabolic pathway of the organism to metabolize the unnatural sugar with bioorthogonal groups to the original position of the natural glycan, and then realize the labeling and research of the unnatural sugar through the bioorthogonal reaction. This method made outstanding contributions to the identification (101) and dynamic changes (101) of glycosylation. Metabolic labeling techniques were also utilized to explore the glycosylation of cell surface glycoproteins. Cell surface glycoproteins are metabolically labeled with functionalized sugars and then labeled with biotin by copper-free click chemistry. Biotin-containing surface glycopeptides were selectively enriched and analyzed with MS. On average, 683 glycosylation sites and 354 surface glycoproteins are identified per cell (102). Glycoproteins were quantified in combination with label-free quantification, distinguishing between cell-specific and cell-ubiquitous glycoproteins (102). This study led to a better understanding of cell surface glycoproteins, and provided important information for the discovery of new biomarkers and drug targets.

Metabolic labeling techniques were also used to visualize, identify, and quantify proteins. The labeling of three carbohydrate analogs (GalNAz, ManNAz and GlcNAz) was compared, and the results showed that GalNAz labelled more cell surface protein glycosylation sites than GlcNAz or ManNAz (102). Not only the metabolic ratios of different sugars on *N*-glycans are different, but also the incorporation efficiency of the same sugar among *N*-/*O*-glycans, glycosides and glycosphingolipids is significantly different (104). A comprehensive, site-specific analysis of changes in *N*-glycosylation of surface proteins on statin-treated versus untreated cells was performed. Compared to untreated cells, many glycopeptides were downregulated in statin-treated HepG2 cells because statins prevented the synthesis of dolichol, which is essential to form dolichol-linked precursor oligosaccharides. *N*-glycosylation on surface proteins associated with Alzheimer’s disease was found to be downregulated (103). Furthermore, with the use of stable isotope labeling of amino acids in cell culture (SILAC), time-dependent changes in cell surface glycoprotein abundance were localized and quantified for the first time (105). Briefly, after cells were subjected to full heavy isotope incorporation and full light isotope incorporation with SILAC method, cell collection was performed every two hours until the 48-hour time course was completed. Over time, heavy proteins are degraded, and newly synthesized proteins are theoretically light proteins, thus measured protein half-life (105, 106). However, a limitation of SILAC method is that proteins with very long half-lives may not be accurately determined because the protein may not be renewed over the course of the assay time. Also, SILAC strategy was used to explore the conversion rate of *O*-GlcNAcylated proteins (107). Glycoproteins on the cell surface are dynamic to adapt to the changing extracellular environment. Thus, the dynamic changes of these glycoproteins can guide disease states with important biomedical significance.

Currently, few unnatural carbohydrate metabolism precursors are widely used, which greatly limits the systematic research of glycosylation in a cell membrane protein (93). Unnatural sugar incorporation relies on competing cellular metabolic processes with natural sugars, which is inefficient and time-consuming. The use of glycosyltransferases and nucleotide-sugar analogs to directly label cell surface glycans was also pursued by researchers (93). Selective exo-enzymatic labeling (SEEL) method was developed to efficiently label cell surface glycans with recombinant sialyltransferases and nucleotide-Sia analogs. Two sialyltransferases, ST6Gal1 and ST3Gal1, were used to label the Sia of *N*- and *O*-glycans, respectively. SEEL in combination with MS identified 37% more sialylated proteins than metabolic labeling methiod. This SEEL study compared the levels of sialylated proteins in undifferentiated vs. differentiated human erythroleukemia cells (HEL), and found that differentiated cells had more *N*-linked sialylated proteins (108). Biotin-functionalized nucleotide-Sia analogs were synthesized to label cell surface Sia in one step, which labels nearly twice as many proteins as the two-step SEEL method. The protocol of this one-step strategy is technically simple, and the transport and turnover of glycoproteins can be easily explored (109), with higher sensitivity compared to typical two-step reporting strategy (110). The glycosyltransferase approach is also flawed and limited to study glycoproteins that can serve as enzyme substrates. However, due to the simple steps and high efficiency, it is especially suitable to detect low-abundance glycan epitopes on living cells.

#### 4.3.3 MS analysis of regional membrane protein glycosylation

Glycoprotein interaction networks are important in many intracellular and extracellular events, and abnormal protein interactions are closely related to various diseases including cancers (14). A proteomic approach in combination with chemical cross-linking, enzymatic reaction, and MS identification was developed to systematically characterize proteins that interact with surface glycans. Bis(sulfosuccinimidyl)suberate (BS^3^), a membrane-impermeable crosslinker, was first used to covalently crosslink surface glycoproteins and their interacting proteins. For the extraction of glycoproteins, a strategy similar to the CSC technique was adopted. Galactose oxidase was used to oxidize glycans on surface glycoproteins, which were then enriched for surface glycoproteins and their interactors by hydrazine chemistry, followed by quantitative proteomics. As a result, it identified more than 300 proteins interacting with surface glycoproteins, and the glycoprotein interaction network was constructed (111). Combined with chemical cross-linking, the analysis of cell surface interaction networks, especially glycan-interacting proteins, became more precise. Azide-labeled Sia on the cell surface through the metabolic pathway, and the bifunctional linker was used to covalently couple Sia and Sia-interacting proteins. Cells were lysed and trypsinized, and the cross-linked glycan-peptides were purified with reversed-phase chromatography columns and strong cation exchange cartridges, and analyzed with reversed-phase liquid chromatography-high-resolution Orbitrap MS. The enriched glycan-peptides were identified by an improved proteomic approach with fragmentation using high-energy collision-induced dissociation. This study unequivocally provides direct information on the network of Sia-mediated protein action on the cell membrane (112). For glycan-protein interactions, covalent bonds created by cross-linking between interacting proteins cannot be cleaved by MS, which is difficult to make direct identification with search software. Proximity labeling-based methods are frequently used to analyze protein interactions; especially APEX2-based methods were widely used in proteomics because of its high catalytic activity, small size (28 kDa), and activity in different cellular compartments (113). APEX2 is fused to the *N*-terminus of Galectin-3 and mapped glycoproteins that interact with Galectin-3. Quantitative proteomics based on tandem mass tag (TMT) labeling identified these interacting glycoproteins with high sensitivity. At the same time, the glycoprotein interacting with galectin-3 was further verified by *in vitro* experiments such as WB (114). In addition to enzymes, chemical probes with catalytic activity are also developed to study lectin-interacting proteins. Iron (S)-1-(p-bromoacetamidobenzyl) EDTA (FeBABE) was used as a catalyst to coupling lectins. Free radicals are generated in the presence of H_2_O_2_ to oxidize lectin-interacting proteins, and lectin-interacting cell surface glycoproteins were identified with MS. This method was extended to study surface glycoproteins that interact with different types of lectins, such as SNA, MAL, AAL, and WGA (115). Similar methods are also developed to identify Sia-interacting proteins (116). Such methods provide an unprecedented insight into the interaction of lectins with specific glycoproteins.

MS has made outstanding contributions to the comprehensive analysis of glycosylation studies of plasma, cell membrane proteins, and glycan-interacting proteins, and the widespread use of MS is mainly due to several key advantages: (i) MS analysis does not require antibodies that are expensive, cumbersome to obtain, and limited in types. Moreover, the performance of antibodies varies to affect the test results. (ii) MS does not require prior knowledge of the type of protein to be studied. WB, immunoprecipitatioin (IP), and other methods commonly used in traditional biology require pre-judgment of the proteins to be analyzed. MS is a common technique in analysis of proteins. Without prior knowledge of proteins, MS enables large-scale marker screening in complex biological samples. (iii) The advent of tagging methods, such as iTRAQ and TMT labeling, can efficiently perform deep quantification of biomarkers, which has an essential feature in comparison of multiple samples. (iv) Perhaps, the most significant advantage of MS-based methods is able to obtain the detailed structural information on glycan structures and to identify glycosylation sites, which might directly impact cell function. In recent years, the combination of chemical biology and MS has made an indelible contribution to the research of glycosylation at different specificity levels, which deepens one’s understanding of the synergistic regulation of cellular protein activities. We believe that it is the focus and direction of future research (**Table 2**).

### 4.4 Fluorescence imaging-based *in situ* cellular glycan analysis

Although proteomics and glycomics have made great advances in the *in vitro* research of glycoglycan structure and function, they cannot provide *in situ* real-time qualitative or quantitative information on glycoglycans on cells, especially the spatial distribution information. Moreover, the complex cleavage and separation process prior to MS analysis might lead to unpredictable loss of glycan information (117). Some studies used lectin, metabolism and other methods to study the overall state of glycosylation of tumor cells (118). In order to achieve more precise analysis, more comprehensive surface accessibility, higher sensitivity, and wider applicability, cell-specific (119–123) and protein-specific (124–128, 132–134) glycan *in situ* analytical methods have been continuously developed in the past five years.

#### 4.4.1 Cell-specific glycosylation analysis

A common method to image glycans of specific cells is to design the caged probes, and this probe can be activated by enzymes that are produced by target cells, such as cancer-associated proteases. A cathepsin B-specific cleavable substrate (KGRR) was conjugated to an azide-modified metabolic sugar precursor, where specific sites of the azide sugar are blocked from being taken up by cells (119). When cathepsin B was present on the surface of tumor cells, it acted like “scissors”, chopping peptide fragments, releasing metabolic precursors, and causing tumor cells to generate unnatural glycans containing azide groups (119). For cell culture and tumor-bearing mice, unnatural glycans on the surface of tumor cells were conjugated to near-infrared fluorescent (NIRF) dye-labeled molecules *via* a bioorthogonal click reaction (119). However, this method is only applicable to a limited number of cells with extracellular protease expression. To overcome this limitation, a metabolic labeling method that target to specific cells was developed by encapsulating unnatural sugars in liposomes modified with the targeting integrin αvβ3. Azide-labeled unnatural saccharides were selectively present in specific cells *via* receptor-mediated endocytosis, followed by coupling to fluorescent dyes *via* copper-free click chemistry (120). This strategy was also extended further to the *in vivo* level, where intravenously injected liposomal nanoparticles selectively bind to cancer cell-specific receptors to install azide into melanoma glycans in a tissue-specific manner (120). Such studies are promising for tumor-specific imaging or drug delivery. Some studies also directly act on the cells with enzymes modified with targeting ligands, and achieve cell selectivity by adjusting the concentration of probes (121). To avoid non-specific reactions caused by collisions, this method must use very low enzyme concentrations, and cell specificity is not optimistic. A strategy was developed to achieve cell-selective glycan remodeling by modulating enzymatically active center accessibility (SEA). Encapsulation of enzymes with MOFs prevents them from reacting with macromolecular enzyme substrates, and the encapsulated enzymes bind to target cells and degrade encapsulates to remodel the target cells. The SEA protocol adopts a modular design and is expected to be a general tool for cell-selective glycan analysis (122). Furthermore, the thermosensitive microgel was used to encapsulate sialidase, combined with the targeted recognition of aptamers, to achieve cell-specific desialylation, and for the first time, tumor-specific desialylation of complex tissue sections was achieved. This method enhances the killing ability of NK cells to target tumor cells through heat-triggered cell-specific desialylation, which provides a new idea for cancer therapeutic intervention targeting glycoimmune checkpoints (123).

#### 4.4.2 Protein-specific glycosylation analysis

Glycans on specific proteins play an important role in regulating the structure and function of proteins to further affect the biological function and physiological state of cells. Therefore, protein-specific glycan analysis provides a powerful tool to reveal glycan-related biological processes. Fluorescence resonance energy transfer (FRET) is the main method to analyze protein-specific glycans (117, 124). Methods based on site-specific duplexed luminescence resonance energy transfer (D-LRET) (125), hierarchical coding (HieCo) (126), localized chemical remodeling (LCM) (127), and DNA enzymatic reactions (128, 132) have also been developed in the past five years. A FRET strategy based on hybridization chain reaction (HCR) amplification was reported (124). Briefly, target cells were subjected to metabolic labeling of glycans to modify FRET donors. Aptamers that can trigger HCR and generate a large number of receptors were added, and the HCR nanoassembly induced the amplification and labeling of the target protein, which resulted in a high FRET signal for enhanced imaging of cell surface glycosylation. To achieve simultaneous imaging of two glycans on a specific protein, upconversion nanoparticles (UCNPs) with multicolor luminescence properties was used as a common donor to construct a D-LRET system on a specific protein on the cell surface (125). Aptamer-modified UCNPs were able to specifically bind to the target MUC1. Meanwhile, two different fluorescent receptors, AF555 and AF660, were labeled with metabolic techniques on two target monosaccharides, Sia and Fuc. Two glycan-labeled fluorescent acceptors on MUC1 were simultaneously excited by D-LRET under near-infrared excitation. This system enabled simultaneous imaging of Sia and Fuc on MUC1 on different cell surfaces. Relative quantification of Sia and Fuc on MUC1 was also achieved with *O*-GalNAc as an internal control (126).

The FRET strategy needs to use two different fluorophores that can undergo fluorescence resonance energy transfer when used for protein-specific glycoform imaging. It is very difficult to obtain two pairs of fluorescent donor-acceptor pairs that do not interfere with each other at the same time, so it is difficult for this strategy to simultaneously image multiple glycans in a specific protein. This problem was addressed by designing a hierarchical coding strategy. DNA sequences were used to encode proteins and different classes of glycans. Decoding process started with the addition of a “time code” that exposed the “protein code”. Exposed “protein code” was hybridized with a hairpin that was covalently modified on the sugar, opening the hairpin and exposing the “monosaccharide code”. The clever introduction of “time encoding” in this strategy strictly distinguishes the encoding and decoding process, and the decoding event can be started at any time (126). A similar method was developed to visualize signal amplification of glycans on specific proteins with metabolic labeling and proximity-induced hybridization chain reaction (133). The number of DNA sequences that could be used as markers was theoretically infinite, so this method, with its ability to image many different glycoforms simultaneously, is a scalable and versatile platform.

LCM strategy was proposed to remodel Gal/GalNAcon-specific proteins. Galactose oxidase activity was inhibited in the presence of potassium ferrocyanide, and galactose oxidase activity was “on” when potassium ferricyanide was added. This switch enabled the specific oxidation of Gal/GalNAc at the end of MUC1 on the cell surface. Bioorthogonal labeling was then performed for the purpose of localized glycan analysis (127). DNA technology was used to design a hairpin structure with filter function for the imaging of specific protein glycoforms on the cell surface. The platform relied on the nicking action of restriction endonuclease (NE), and the two ends of the designed hairpin molecular probe were modified with fluorophore and quencher, respectively (128). The site specifically recognized by NE was designed in the middle region of the hairpin. The probe was not cleaved by NE in the hairpin configuration, but could be cleaved by NE when the hairpin was opened by the protein probe. Fluorescent signal of the closed-loop structure was covalently retained at the end of the sugar chain, and the fluorescent signal of the open-loop structure was released into the solution. Based on this method, a multifunctional DNA localization nanomachine was further improved and developed to analyze multiple targeted modifications (MOIs) at the protein-specific level (132).

In summary, it clelarly demonstrates that the current protein-specific glycoform imaging strategies need to use a “gating” guarantee mechanism. For example, the FRET strategy relies on distance as the “gating”, the LCM strategy uses ions as the “gating”, and the HieCo strategy designs DNA as the “gating”. These strategies are cleverly designed, but also suffer from disadvantages such as limited generalizability (LCM) and complex design (HieCo). Therefore, a fundamental shift in detection mode is also required in the future to achieve highly sensitive protein-specific glycoform imaging with a simplified “gated-free” design (**Table 2**).

## 5 Glycan-related tumor therapy

### 5.1 Glycan-related targeted therapy

As previously discussed, tumor-associated glycans/glycoproteins have been widely used as biomarkers for clinical diagnosis and prognostic assessment of patients (133). The abnormal changes in glycosylation on the surface of tumor cells relative to normal cells can also be extended to therapeutic targets (134). In the context of 3P medicine, abnormal glycosylation provides a clear label for tumor cell identification, and individualized diagnosis and treatment plans can be implemented according to the special situation of each patient (135). It can greatly improve the specificity of tumor treatment and has a huge impact on monitoring and treatment. Research on targeting glycosylation mainly covers two levels:

(i) tumor suppressive effects of glycan-binding molecules (136–138). Since cancer-specific glycan biomarkers are only highly present on the surface of tumor cells, glycan-binding molecules can be used to discriminate tumor cells from normal cells. The glycan-binding molecule contains lectins and anti-glycan antibodies. The addition of the glycan-binding molecule blocks the corresponding signaling pathway and triggers cancer cell death. For example, the ganglioside focusyl-GM1 is a tumor-associated antigen that is aberrantly expressed in human small cell lung cancer but not in most normal adult tissues, making it a promising target in tumors (136). A new fully human anti-focusyl-GM1 antibody was discovered. In multiple mouse SCLC models, the focusyl-GM1 antibody showed good efficacy and was well tolerated. The focusyl-GM1 antibody was used in a preclinical model of small cell lung cancer and showed strong *in vitro* and *in vivo* antitumor activity. As a common tumor biomarker, GD2 antibody was also used in the treatment of neuroblastoma (137). It was found that interleukin 2 in combination with anti-GD2 antibody therapy improves outcomes of high-risk neuroblastoma patients who respond to standard induction and consolidation therapy. The GM3 (Neu5Gc) ganglioside, a tumor-specific antigen, was identified as a promising target for cancer immunotherapy (138). A humanized antibody against this ganglioside, 14F7hT, was developed and demonstrated to have significant antitumor effects. Studies have shown that 14F7hT has antibody-dependent cytotoxicity and anti-tumor effects *in vivo*.

(ii) Targeting function of glycan-binding molecules (139–149). Glycan-binding ligands can be used to increase the selectivity and efficiency of antineoplastic drugs against cancer cells and increase their concentration at tumor sites. For example, Tn antigens are highly specific to tumors, and Tn antibodies have been used in targeted drug delivery (139). With the use of the specificity of Tn monoclonal antibodies and the strong cytotoxicity of anticancer drugs, a new type of antibody-drug targeting Tn antigens was introduced. The specificity of monoclonal antibody *in vivo* was first explored, and the antibody-drug conjugate was further applied to Tn-positive tumor cells *in vitro*, showing effective cytotoxicity, and the cytotoxicity was positively correlated with the expression level of Tn in the tumor. Antibody-drug conjugates also showed potent antitumor activity *in vivo*. The study demonstrated for the first time the effectiveness of Tn antibodies as antibody-drug conjugates. Further, Tn antibody was used to provide targeting effect for the drug-encapsulated nanocapsules, the encapsulation efficiency of nanocapsules for antitumor drugs reached 99.9% and the biotin-avidin method was used to attach antibodies to nanocapsules (140). *In vitro* uptake studies and viability assays in the A549 human lung cancer cell line demonstrated that Tn antibodies enhanced nanoparticle internalization and decreased cell viability. MUC1 is aberrantly expressed in epithelial malignant cells, making it an interesting diagnostic and therapeutic target (141). NK cells express abundant Fc receptors, which promote the binding of NK cells and tumor cells, thereby enabling NK cells to precisely target and destroy cancer cells. A protein coupled with MUC1 antibody and Fc receptor was developed, which was highly specific to tumors, and applied to tumor immunotherapy. In addition to antibodies, aptamers are also widely used for targeted binding of proteins due to their small molecular weight and high specificity (142). A novel MUC1 aptamer-modified nanocomplex was developed for the targeted delivery of epirubicin (143). In addition to chemotherapeutic drug delivery, the study of MUC1 aptamer-functionalized hybrid nanoparticles for targeted delivery of miRNA-29b to non-small cell lung cancer was proposed (144). The MUC1 aptamer was coupled to the nanoparticle surface and enhanced the selectivity of miRNA-29b for tumor cells and tissues. Sialic acid and fucose are typical tumor cell biomarkers that have been widely used in tumor-targeted therapy (145). Bispecific Janus agglutination can simultaneously bind sialic acid and fucosylated glycoconjugates, and Janus lectin-mediated targeted tumor therapy was developed (146). Janus lectins were used to modify giant unilamellar vesicles to induce lipid internalization, leading to precise drug uptake by human epithelial cancer cells. In addition to macromolecular proteins, small molecules such as phenylboronic acid can also specifically bind sialic acid (147). Boronic acid-targeting sialic acid nanocomposites were proposed for the combined delivery of etoposide and the herbal berberine for local treatment of lung cancer. Etoposide, as a potential lung cancer therapy, is limited in its application due to its poor solubility and overall side effects. This study shows that the inhalable nanocomposite has a good antitumor effect. SLea functionalization is a glycan known to mediate tumor metastasis (148). Monoclonal antibody-targeted nanoparticles targeted the controlled release of cytotoxic drugs for intravenous and oral support. The nanocapsules also reduced the initial toxicity of the drug to gastric cells. The study of CD19 antibody chimeric CAR T cells for targeted immunotherapy of tumors resulted in remission in most refractory and relapsed patients (149).

Altered glycosylation is a key change in tumors, and aberrant glycosylation is a component of tumor growth, survival, metastasis, and immune evasion (150). Targeting glycosylation has many advantages in tumor 3P medicine. Cancer-associated glycans represent a valuable opportunity for cancer diagnosis, prognosis, and treatment. Molecules exhibiting glycan recognition properties, as described herein, may represent a powerful strategy for stem cancer diagnosis and treatment (151, 152).

### 5.2 Glycan-related vaccine design and immunotherapy

Immune checkpoint inhibitors, such as anti-PD1 and anti-CTLA-4 blocking antibodies, were widely used in tumor therapy and improved long-term survival of patients (153, 154). However, not all patients benefit from checkpoint inhibitor therapy. The reason for these lack of benefit is often the absence of T-cell infiltration in the tumor microenvironment, a subset of patients who could benefit from tumor vaccination (154, 155). Antitumor vaccines can be divided into preventive tumor vaccines and therapeutic tumor vaccines. Preventive tumor vaccines are represented by the HPV (human papillomavirus) vaccine approved by the FDA in 2006 (136). The HPV vaccine was developed by Merck & Co., which is immunized to healthy people before the occurrence of tumors, and the human body obtains tumor immune responses in advance (156). The HPV vaccine can effectively prevent the occurrence of cervical cancer in women, and has achieved great clinical success. Therapeutic tumor vaccines are injected after the occurrence of tumors. It is hoped that the vaccines can induce specific anti-tumor immune responses in tumor patients to achieve the purpose of tumor treatment. Therapeutic tumor vaccines developed with glycoconjugates are a research hotspot and are regarded as an innovative biologic that can be used in a variety of therapeutic settings. Glycoconjugate vaccines are divided into three categories: (i) glycolipid antigens, such as gangliosides Fuc-GM1 (157), GM3 (158, 159), GD2 (160), GD3 (161, 162), SLeA (163), SLeX (163), and Globo H (164). (ii) glycoproteins, such as mucin-associated epitopes Tn, TF, and STn (62, 165); (iii) proteoglycans, such as polysialic acid (147). Glycoconjugate vaccines have high efficiency, low toxicity and high specificity, and are a hot spot in tumor immunotherapy (**Table 4**).

**Table 4:** Representative polysaccharide vaccine in tumor immunotherapy.

Tumor-associated polysaccharide vaccines		Structure	Cancer type	References
Type	Glycan			
Ganglioside	Fuc-GM1	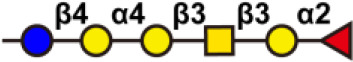	Liver cancer, lung cancer	(156)
	GM3	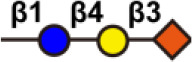	Lung cancer, brain cancer, breast cancer, and melanoma	(157, 158)
	GD2	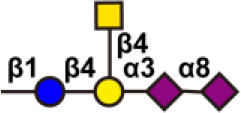	Neuroblastoma, lymphoma, melanoma, and osteosarcoma	(159)
	GD3	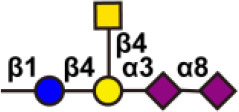	Breast cancer, melanoma	(160, 161)
	SLeA		Colon, stomach, biliary, and pancreatic cancer	(162)
	SLeX	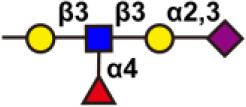		
	Globo H	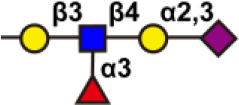	Small cell lung, prostate, pancreatic, gastric, and ovarian cancers	(163)
Glycoproteins	Tn	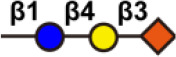	Bladder, colorectal, ovarian, and breast cancer	(164, 165)
	STn	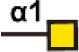		
	TF	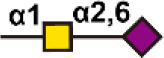		
Proteoglycan	Polysialic acid	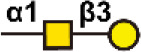	Lung cancer, breast cancer, and neuroblastoma	(62)

## 6 Future perspective

### 6.1 Glycosylation analysis and 3P medicine

Tumor-related glycosylation changes are a significant feature of cancer diagnosis and prognosis. In recent years, researchers have made great efforts to better analyze and utilize glycosylation. For glycosylation analysis, methods including immunochemical methods, lectin recognition-based methods, MS-related methods, and fluorescence imaging-based *in situ* analysis methods have been developed. Immunochemical methods have high sensitivity and specificity, but are overly antibody-dependent and prone to false positives. Lectin microarray is a fast, sensitive, high-throughput glycan analysis technique suitable for studies with large sample sizes. Immunochemical methods, lectin recognition-based methods, and fluorescence imaging-based *in situ* analysis methods all require prior knowledge of the protein being analyzed, which is disadvantageous for 3P medicine PPPM (166). MS is a versatile technique that enables large-scale marker screening in complex biological samples without prior knowledge of proteins, enabling detailed structural information about glycan structure, and identifying potential implications for cellular function. Traditional MS technology cannot perform *in situ* analysis at different specificity levels. The combination of chemical biology methods with MS technology makes up for this drawback. A thorough investigation of a patient’s inability to undergo glycosylation at different levels of specificity can help map precise glycophenotypes (167).

### 6.2 Glycan-related immunotherapy and 3P medicine

Cell surface glycans are key cellular components that influence cell recognition behavior. Injecting glycan-related vaccines often brings problems of poor specificity, which is not conducive to 3P medicine. Glycan editing on the cell surface to achieve glycoform remodeling and further modification of other biomolecules can modulate cellular recognition and communication functions. Using glycan editing to enhance anti-tumor immune responses by blocking glycan immune checkpoints has brought new breakthroughs in the field of cancer therapy (121, 168, 169). Sia residues send a healthy signal to the body, suppressing immune activation through multiple pathways. The high sialylation status of tumor cells/tissues plays an important role in the ability to evade immune recognition. Sia upregulation has been associated with poor tumor prognosis and decreased immunogenicity. Removal of cell surface Sia was found to enhance NK cell activation. An antibody-enzyme conjugate biotherapeutic molecule was designed (121). The antibody specifically recognizes tumor cells, guides sialyl cleavage enzymes to specifically desialylate tumor cells, and guides immune cells to kill desialylated cancer cells. The conjugate increased tumor cell killing compared to the antibody alone. This method was successfully used in breast cancer mice (168). However, this method lacks cell-specific controls, thermosensitive smart microgels were used to modulate the editing ability of glycan editing enzymes, and in combination with aptamers, an *in situ*, cell-specific glycan editing strategy was developed. Sia cleavage was limited to the surface of the target cell, thus enabling thermoresponsive cell-specific glycan editing. This method achieves the enhancement of innate anti-tumor immunity and avoids the interference of glycan editing with the normality of other cells. Redirecting or boosting the immune response is an effective treatment (118). A key challenge of this immunotherapy is to selectively install molecules that recruit immune responses on the surface of relevant cells. By selectively delivering metabolic sugar precursors to folate receptor-overexpressing cells, an azide group was added to cell surface glycans. Rhamnose was efficiently introduced to the surface of expressing cells. Studies have demonstrated that rhamnose mounted on the target surface recruits anti-rhamnose antibodies and promotes apoptosis of folate receptor overexpressing cells through complementation-dependent cytotoxicity (CDC) and antibody-dependent phagocytosis (ADCP) (169, 170). In turn, customized treatment algorithms can be created to provide optimal clinical approaches for personalized, predictive and preventive medical services, which we believe will be the focus and direction of future research.

## 7 Conclusion and expert recommendation in framework of 3P medicine in cancer

Glycosylation modification is one of the most important post-modification modifications of proteins. Glycans greatly enrich the biological information of proteins, thereby enhancing their role in cellular behavior. Glycosylation is a template-free process, and the expression of related genes is affected by transcription factors, epigenetic changes, microenvironment, etc. The complexity of carbohydrates and the limitations of research methods make the research of glycomics seriously lag behind the research of genomics and proteomics. In the past five years, driven by the latest technological advancements, analytical methods based on immunochemical methods, lectin recognition, MS, and fluorescence imaging have gained momentum in cancer research, in terms of providing new glycosyl-based markers, has considerable potential. Although immunochemical-based methods are limited by antibody species and affinity, assays for specific glycosylated target proteins are easier to standardize and reduce redundancy, making them suitable for clinical applications. The *in situ* analysis methods based on fluorescence imaging have made great efforts to study the dynamic changes and spatial distribution of glycans and functions; however, it cannot obtain specific glycan structures. A significant advantage of MS-based method is the ability to obtain detailed information about glycan structure and to identify glycosylation sites that may have direct effects on cellular function. However, they cannot provide real-time information about glycoglycans on intact cells, especially the spatial distribution. The organic combination of chemical biology and MS has made an indelible contribution to the research of glycosylation at different levels of specificity, deepening our understanding of the synergistic regulation of cellular activities by proteins. Combining biochemical, *in situ* analysis, and omics techniques to research glycosylation together provides interdisciplinary insights into deciphering diseases at multiple levels, which we believe is also the focus and direction of future research. In addition, glycans in organelles, such as mitochondria, Golgi and nucleus, need more attention. At the same time, recent research results have also highlighted the relationship between glycosylation and immunity, suggesting that the use of glycan editing can enhance anti-tumor immune responses, which has brought new breakthroughs in the field of cancer therapy. In the future, glycosylation will surely be revealed with new diagnostic, prognostic, and even therapeutic applications. After entering the new century, medicine has entered a new 3P era, which represents the ultimate goal and highest stage of medical development. Glycosylation-based marker analysis and immunotherapy have achieved rapid development in the past decade. This new model of early warning, prevention and individualized treatment has also promoted the rapid development of oncology to certainly improve people’s quality of life.

## Author contributions

YG collected and analyzed literature, designed and wrote the manuscript. WJ and JY participated in the collection and analysis of literature. XZ conceived the concept, coordinated, critically revised manuscript, and was responsible for the corresponding works. All authors approved the final manuscript.

## Funding

This work was supported by the Shandong First Medical University Talent Introduction Funds (to YG), the Shandong First Medical University Talent Introduction Funds (to XZ), Shandong First Medical University High-level Scientific Research Achievement Cultivation Funding Program (to XZ), the Shandong Provincial Natural Science Foundation (ZR202103020356 or ZR2021MH156 to XZ), and the Academic Promotion Program of Shandong First Medical University (2019ZL002).

## Conflict of interest

The authors declare that the research was conducted in the absence of any commercial or financial relationships that could be construed as a potential conflict of interest. 

## Publisher’s note

All claims expressed in this article are solely those of the authors and do not necessarily represent those of their affiliated organizations, or those of the publisher, the editors and the reviewers. Any product that may be evaluated in this article, or claim that may be made by its manufacturer, is not guaranteed or endorsed by the publisher.

## Glossary

AAL: Aleuria aurantia lectin
ADCP: antibody-dependent phagocytosis
AFP-L3: fucosylated α-fetoprotein
AGP: α-1-acid glycoprotein
Asn: asparagine
BH: biotin hydrazine
CDC: complementation-dependent cytotoxicity
CSC: cell-surface-capturing
CSPA: Cell Surface Protein Atlas
D-LRET: duplexed luminescence resonance energy transfer
EGFR: epidermal growth factor receptor
ELISA: enzyme-linked immunosorbent assay
ER: endoplasmic reticulum
ESI: electrospray ionization
FRET: fluorescence resonance energy transfer
Fuc: fucose
Gal: galactose
GalNAc: N-acetylgalactosamine
Glc: glucose
GlcA: glucuronic acid
GlcNAc: N-acetylglucosamine
HCR: hybridization chain reaction
HEL: human erythroleukemia cells
HieCo: hierarchical coding
HPV: human papillomavirus
IDOA: iduronic acid
L-ELISA: lectin-antibody sandwich enzyme-linked immunosorbent assay
LCM: localized chemical remodeling
MAL: Maackia amurensis leukoagglutinin
MALDI: Matrix-assisted laser desorption/ionization
Man: mannose
MOF: metal-organic frameworks
MOIs: multiple targeted modifications
MS: mass spectrometry
NCAM1: neural cell adhesion molecule 1
NE: nicking action of restriction endonuclease
NIRF: near-infrared fluorescent
PNGase F: peptide-N-glycosidase F
PTMs: post-translational modifications
Ser: serine
Sia: sialic acid
SILAC: stable isotope labeling of amino acids in cell culture
SEEL: selective exo-enzymatic labeling
SLea: sialyl-Lewis A
SLex: sialyl-Lewis X
SNA: Sambucus nigra agglutinin
Thr: threonine
TMT: tandem mass tag
TOF: time-of-flight
UCNPs: Upconversion nanoparticles
WB: Western blotting
WGA: Wheat germ agglutinin
Xyl: xylose
